# Network analysis of mitonuclear GWAS reveals functional networks and tissue expression profiles of disease-associated genes

**DOI:** 10.1007/s00439-016-1736-9

**Published:** 2016-10-04

**Authors:** Simon C. Johnson, Brenda Gonzalez, Quanwei Zhang, Brandon Milholland, Zhengdong Zhang, Yousin Suh

**Affiliations:** 1 Department of Genetics, Albert Einstein College of Medicine, Bronx, NY USA; 2 Department of Ophthalmology and Visual Sciences, Albert Einstein College of Medicine, Bronx, NY USA; 3 Department of Medicine, Endocrinology, Albert Einstein College of Medicine, Bronx, NY USA

## Abstract

While mitochondria have been linked to many human diseases through genetic association and functional studies, the precise role of mitochondria in specific pathologies, such as cardiovascular, neurodegenerative, and metabolic diseases, is often unclear. Here, we take advantage of the catalog of human genome-wide associations, whole-genome tissue expression and expression quantitative trait loci datasets, and annotated mitochondrial proteome databases to examine the role of common genetic variation in mitonuclear genes in human disease. Through pathway-based analysis we identified distinct functional pathways and tissue expression profiles associated with each of the major human diseases. Among our most striking findings, we observe that mitonuclear genes associated with cancer are broadly expressed among human tissues and largely represent one functional process, intrinsic apoptosis, while mitonuclear genes associated with other diseases, such as neurodegenerative and metabolic diseases, show tissue-specific expression profiles and are associated with unique functional pathways. These results provide new insight into human diseases using unbiased genome-wide approaches.

**Electronic supplementary material:**

The online version of this article (doi:10.1007/s00439-016-1736-9) contains supplementary material, which is available to authorized users.

## Introduction

**Fig. 1:**
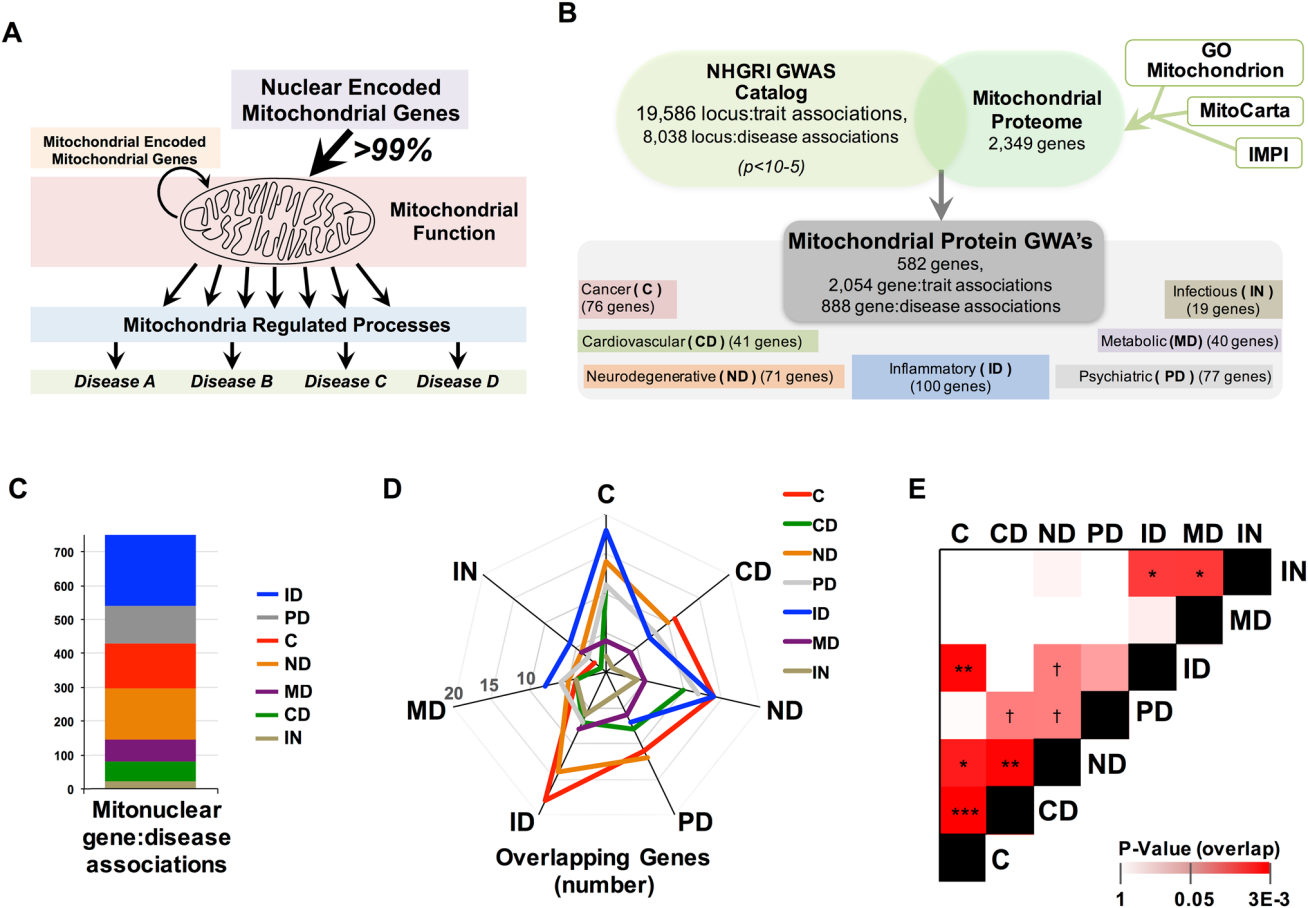
Nuclear encoded mitochondrial proteins associated with human disease by GWAS. **a** The majority of mitochondrial proteins are encoded by the nuclear genome and genetic variation in both the nuclear or mitochondrial genome influences disease risk. **b** The mitochondrial proteome was cross-referenced against the entire human GWAS catalog and mitonuclear gene:disease associations were analyzed by disease type. **c** Mitonuclear genes are strongly enriched for GWAS disease associations compared to the whole protein-coding genome. **d** Overlapping genes between each of the disease groups and **e** statistical assessment of the similarity (overlap) of mitonuclear gene signatures between diseases by hypergeometric distribution (see “Methods”, see also Fig. S1). ^†^ *p* = 0.05, *p < 0.05, **p < 0.005, ***p < 0.0005 (*C* cancer, *ID* inflammatory disease, *ND* neurodegenerative disease, *MD* metabolic disease, *CVD* cardiovascular disease)

Mitochondrial dysfunction has been implicated in a broad range of human pathologies from neurodegenerative and cardiovascular diseases to inflammatory disorders, cancer, and aging (Ajith and Jayakumar 2014; Bonomini et al. 2015; Coskun et al. 2012; Goncharov et al. 2015; Lane et al. 2015; Luo et al. 2015; Passos and Zglinicki 2012; Song et al. 2015; Wallace 2012). Mitochondrial function depends on proteins encoded by genes in both mitochondrial DNA (mtDNA) and the nuclear genome (composed of nuclear DNA, nDNA). The mitochondrial genome contains only 13 protein-coding genes, all of which are involved in electron transport chain (ETC) function and account for less than 1 % of the total mitochondrial proteome (mitoproteome), while nuclear genes encoding mitochondrial proteins (mitonuclear genes) are responsible for the remaining >99 % of mitochondrial proteins (Calvo et al. 2016; Pagliarini et al. 2008; Taanman 1999) (Fig. 1a). Rare mutations in mitonuclear genes or mitochondrial DNA resulting in strong defects are known to cause human mitochondrial disorders, but the role of common genetic variation in mitonuclear genes in complex diseases is less straightforward. In particular, relationships between mitonuclear genes identified by genome-wide association studies (GWAS) and human diseases are often unclear. GWAS associations identify disease-associated variation and genetic loci of interest but alone cannot reveal the directional impact of identified variation on gene product expression or function and lack mechanistic or network setting. Accordingly, GWAS provide lists of candidate genes but determining biological context requires further study.

To better define the role of mitochondria in human disease we examined common genetic variation in mitonuclear genes in the context of human diseases using the National Human Genome Research Institute (NHGRI) genome-wide association study (GWAS) catalog. We found that major disease groups (cancer, cardiovascular disease, neurodegenerative disease, metabolic disease, inflammatory disease, psychiatric disorders, and infectious disease) are associated with unique mitochondrial pathway signatures, characterized by distinct protein–protein interaction networks based on STRING analysis as well as unique ontology sets. Genome-wide RNA sequencing expression data from 32 human tissues indicated unique tissue-specific expression profiles for each disease-associated mitonuclear gene group. Finally, examination of GWAS risk alleles using eQTL data revealed the directional impact of genetic perturbations on functional pathways, in addition to individual genes, and provides a model for the contextual role of mitonuclear GWAS risk alleles in cancer. These unbiased genome-wide assessments provide new insights into the pathway and tissue-specific roles of mitochondria in human diseases.

## Results

### A comprehensive mitonuclear GWAS catalog of human disease traits

To examine the role of common genetic variation in mitonuclear genes in human disease, we first compiled a comprehensive mitochondrial proteome using three resources: MitoCarta (Pagliarini et al. 2008), Integrated Mitochondrial Protein Index (IMPI) (Smith and Robinson 2015), and the Gene Ontology (GO) Mitochondrion catalogue (Ashburner et al. 2000) (Fig. 1b). This combined list represents all 2349 genes encoding products demonstrated or predicted to localize to mitochondria (Supplemental File 1). Using the GWAS catalog (Welter et al. 2014) we extracted a total of 19,586 locus:trait associations at *p* value <1 × 10^−5^ that include disease traits, such as cardiovascular disease and cancer, and non-disease traits, for example, hair color or political preferences. We curated this catalog to extract only disease-associated traits, resulting in 8038 locus:disease associations.

We next cross-referenced this catalog against the mitochondrial proteome, using the nearest-neighbor SNP–gene assignments in the GWAS catalog, yielding 2055 mitonuclear locus:trait associations, including 888 mitonuclear locus:disease associations, arising from 583 unique mitonuclear genes (both trait lists provided in Supplemental File 2). Individual gene:disease associations were assigned to the following disease groups for further analysis: cancer, cardiovascular disease, neurodegenerative disease, metabolic disease, inflammatory disease, psychiatric disorders, and infectious disease (Fig. 1b) (Supplemental File 2, Supplemental File 3). Importantly, no mtDNA-encoded genes appear among these locus:trait associations, limiting all analyses to nuclear genes encoding mitochondrial proteins (mitonuclear genes.

### Disease risk gene overlap

Pairwise comparisons of each disease group indicated significant overlaps of associated genes between some disease pairs (Fig. 1d, e). In a recent study comparing similar disease groups using the whole genome, rather than just mitonuclear genes, we found cardiovascular and metabolic diseases share the most genes, while cancer and neurodegenerative disease show the least overlap (Johnson et al. 2015). In contrast, among mitonuclear genes, cancer and neurodegenerative diseases show a high degree of overlap, while cardiovascular and metabolic diseases have only modest overlap (Fig. 1d, e; Fig. S1, see also Supplemental File 3). Significant overlap between disease pairs suggests common mitochondrial mechanisms underlying genetic risk associations.

### Enrichment of protein–protein interactions in mitonuclear gene disease networks

**Fig. 2:**
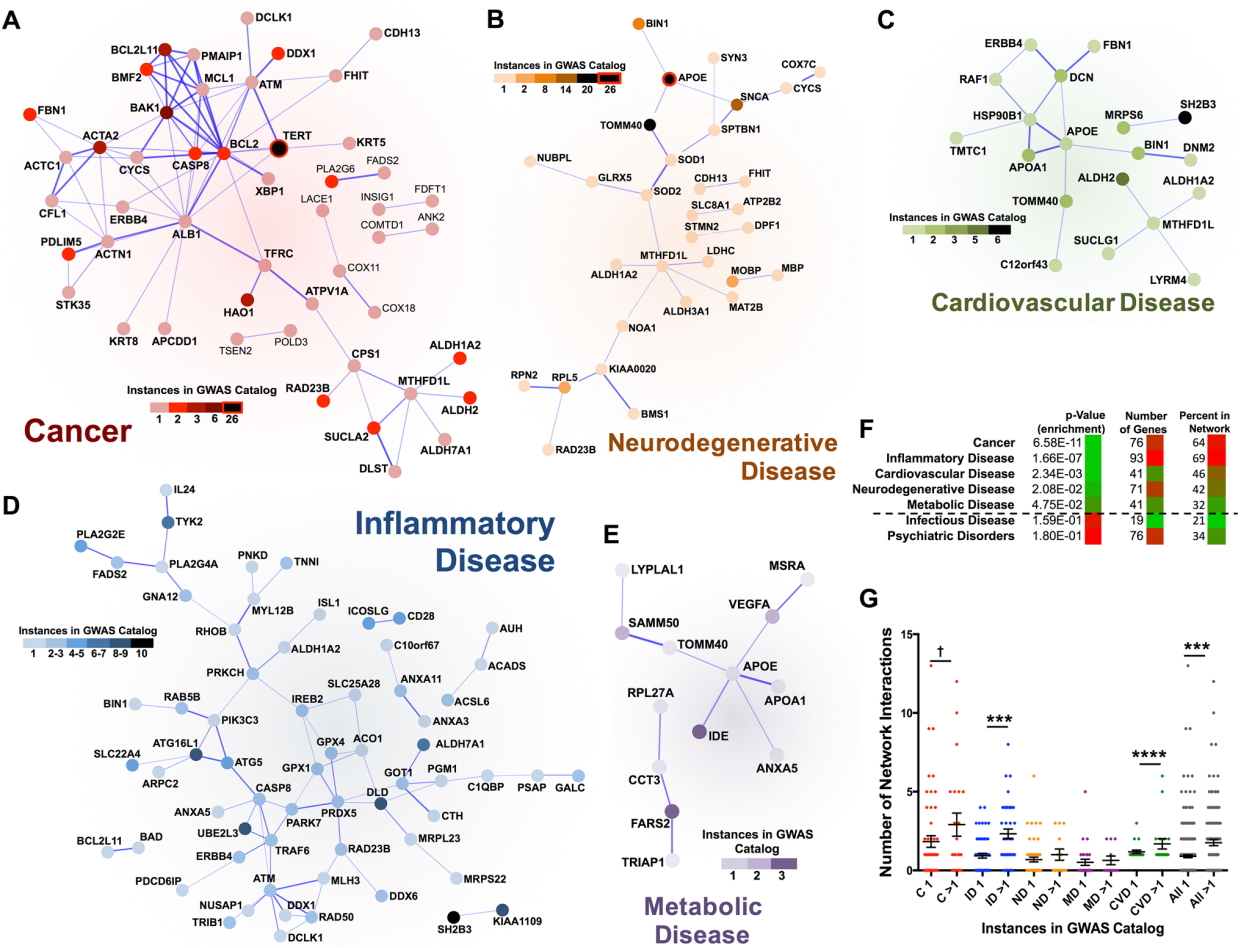
Mitonuclear gene groups associated with disease in GWAS are significantly enriched for protein–protein interactions. STRING networks generated from the gene products of mitonuclear genes associated with **a** cancer, **b** inflammatory disease, **c** neurodegenerative disease, **d** metabolic disease, and **e** cardiovascular disease. Number of disease-specific GWAS catalog appearances indicated by the *shade* of the network symbol. Isolated gene products with no network interactions omitted from these plots. **f** Total genes in each disease group, percentage present in each interaction network, and *p* values for enrichment of protein–protein interactions by STRING pathway analysis. Infectious disease and psychological disorders were not enriched for protein–protein interactions (see Fig. S2). **g** Number of network interactions for each mitonuclear gene appearing once or more than once in the GWAS catalog by disease group; ***p < 0.0005, ****p < 0.0001, ^†^ *p* = 0.15 by pairwise *t* test. (see Supplemental File 4) (*C* cancer, *ID* inflammatory disease, *ND* neurodegenerative disease, *MD* metabolic disease, *CVD* cardiovascular disease)

Since gene products exert their functions through interactions with other cellular components, the impact of a genetic perturbation can spread along the links of any functional network the gene product is involved in, disrupting network function (Barabasi et al. 2011). To investigate the network context of the mitonuclear gene sets associated with human disease, we analyzed each disease group using the Search Tool for the Retrieval of Interacting Genes/Proteins (STRING) (Jensen et al. 2009), a database of known and predicted protein–protein interactions. We found each disease gene set to associate with unique protein–protein interaction network with varying degrees of interconnectivity and network interaction enrichment (Figs. 2, S2). The networks for cancer, inflammatory disease, neurodegenerative disease, metabolic disease, and cardiovascular disease are significantly enriched for protein–protein interactions compared to the mitonuclear gene/GWAS background gene set, the most stringent approach (Fig. 2a–f, see “Methods”), while psychiatric disorders and infectious disease are not (Fig. S2a, b, Supplemental File 4). Notably, the degree of network interaction enrichment was not simply a result of the overall number of GWAS-identified genes in each group, i.e., cancer and psychiatric disorders had the same number of GWAS-identified genes but the cancer group was highly enriched for protein–protein interactions while psychiatric disorders group showed no interaction enrichment (Figs. 2f, S2c).

### Degree centrality of mitonuclear disease genes is associated with frequency in GWAS

The enrichment of unique protein–protein interaction networks among the major disease groups is consistent with a model where complex diseases are driven by the impact of gene products on functional networks. This model suggests that gene products centrally involved in key functional networks might have stronger phenotypic effects, through their many network interactions, and would consequently be associated with stronger genetic associations and appear more frequently in GWAS. Indeed, genes with the highest number of GWAS instances in cancer, inflammatory disease, and neurodegenerative disease also tend to be the most highly interconnected within their respective networks (Fig. 2). To examine the correlation between GWAS hotspots and network connectedness, we analyzed the number of network interactions (degree centrality) of mitonuclear genes appearing only once in the GWAS catalog for a given disease group, compared to those appearing multiple times (Figs. 2g, S2d). As a network-based assessment would predict, genes appearing more than once in the GWAS catalog do have more network interactions than those appearing only once. Cardiovascular disease, inflammatory disease, and the complete mitonuclear gene set show a statistically significantly enrichment for network interactions among genes appearing more than once in the GWAS catalog compared to those with only one GWAS occurrence. Other networks show only modest trend, although these analyses are undoubtedly limited in statistical power by the size of the datasets available.

### Mitochondrial pathways of disease

We next searched for the enriched Gene Ontology (GO) terms for each disease group using the mitonuclear gene GWAS set as background (the most stringent approach). We found that the cardiovascular disease set is significantly enriched (*p* < 0.05 after FDR multiple testing correction) for five terms (Table 1): lipoprotein particle binding, sterol binding, cholesterol binding, lipoprotein particle receptor binding, and alcohol binding. Inflammatory disease set is significantly enriched in 44 terms, which primarily associated with wound healing, stress response, immune responses, and cellular proliferation. Metabolic disease is significantly enriched for β-amyloid binding, cellular component biogenesis, and response to stimulus. No term from the neurodegenerative disease group achieved FDR-corrected significance, but those reaching nominal significance *p* < 0.05 include lipoprotein binding, tau binding, and terms associated with myelin, intracellular trafficking, and calcium (see also Supplemental File 4).

**Table 1:** Gene ontology terms enriched in disease-associated mitonuclear gene sets

GO	Term	*p* value	FDR *p* value
Cancer			
BP	Intrinsic apoptotic signaling pathway	2.26E−07	2.97E−03
BP	Response to external biotic stimulus	7.33E−07	2.97E−03
BP	Response to other organism	7.33E−07	2.97E−03
BP	Response to biotic stimulus	8.83E−07	2.97E−03
BP	Cell type-specific apoptotic process	1.27E−06	3.41E−03
BP	Intrinsic apoptotic signaling pathway in response to DNA damage	2.34E−06	5.24E−03
BP	Positive regulation of mito. outer mem. perm. involved in apoptotic signaling	3.98E−06	7.64E−03
BP	Response to virus	8.46E−06	1.42E−02
BP	Regulation of mito. outer mem. perm. involved in apoptotic signaling	1.50E−05	2.18E−02
BP	Digestive system development	1.95E−05	2.18E−02
BP	Positive regulation of protein insertion into mito. mem. in apoptotic signaling	1.95E−05	2.18E−02
BP	Regulation of proteasomal ubiquitin-dependent protein catabolic process	2.10E−05	2.18E−02
BP	Anatomical structure formation involved in morphogenesis	3.71E−05	3.56E−02
Metabolic disease			
MF	Beta-amyloid binding	6.56E−06	2.58E−02
BP	Positive regulation of cellular component biogenesis	3.57E−06	3.11E−02
BP	Response to light stimulus	4.63E−06	3.11E−02
Cardiovascular disease			
MF	Lipoprotein particle binding	6.56E−06	1.29E−02
MF	Sterol binding	1.37E−05	1.35E−02
MF	Cholesterol binding	1.37E−05	1.35E−02
MF	Lipoprotein particle receptor binding	2.59E−05	2.04E−02
MF	Alcohol binding	7.37E−05	4.83E−02
Inflammatory disease*			
BP	Intracellular signal transduction	6.51E−08	8.75E−04
BP	Response to stress	4.68E−07	3.15E−03
BP	Wound healing	7.25E−07	3.19E−03
BP	Defense response	9.49E−07	3.19E−03
BP	Response to wounding	2.02E−06	5.44E−03
BP	Regulation of body fluid levels	4.58E−06	8.09E−03
BP	Regulation of intracellular signal transduction	5.62E−06	8.09E−03
BP	Regulation of multicellular organismal process	5.71E−06	8.09E−03
BP	Blood coagulation	6.59E−06	8.09E−03
BP	Coagulation	6.59E−06	8.09E−03
BP	Regulation of defense response	7.38E−06	8.09E−03
BP	Positive regulation of multicellular organismal process	7.66E−06	8.09E−03
BP	Hemostasis	7.93E−06	8.09E−03
BP	Regulation of protein metabolic process	8.42E−06	8.09E−03
BP	Regulation of primary metabolic process	1.46E−05	1.31E−02
Neurodegenerative disease^†^			
MF	Tau protein binding	3.37E−05	ns
MF	Structural constituent of myelin sheath	1.06E−03	ns
MF	Phospholipase binding	1.06E−03	ns
MF	Lipoprotein particle binding	3.11E−03	ns
MF	Kinesin binding	6.08E−03	ns
MF	Ankyrin binding	9.92E−03	ns
MF	Calcium ion binding	3.90E−02	ns
MF	Calcium-dependent protein binding	4.02E−02	ns
MF	Copper ion binding	4.81E−02	ns

*ns* not significant

* Truncated at 15 most significant terms

^†^Nominally significant terms

The cancer gene set was the most striking in both the STRING network and GO analyses. In cancer, protein–protein interactions are highly enriched: 84 observed compared to 34 expected, with an enrichment *p* value of 6.58 × 10^−11^. A cluster of highly interconnected factors is readily apparent, composed of well-known tumor suppressors and proto-oncogenes involved in apoptosis including BCL-2, BAK, TERT, and CASP8 (Hassan et al. 2014; Shortt and Johnstone 2012) (Fig. 2a). In addition to the cluster of apoptosis-regulating genes in the STRING network, GO analysis of the cancer mitonuclear gene set revealed significant enrichment of terms related to the regulation of apoptosis (Table 1). Importantly, intrinsic apoptosis, involving mitochondrial outer membrane permeabilization as a mediator of cell death signaling, is specifically enriched, and the most significant GO term is ‘intrinsic apoptotic signaling pathway’.

### Tissue-specific expression of disease-associated mitonuclear gene sets

**Fig. 3:**
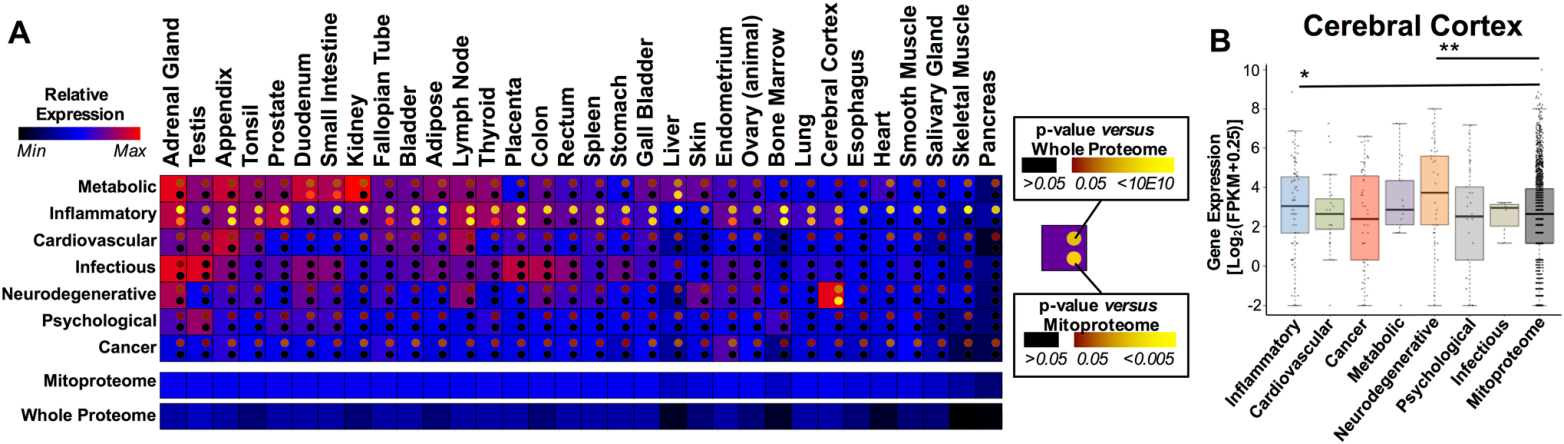
Mitonuclear disease gene sets have unique tissue expression patterns. **a** Relative median expression of each mitonuclear disease gene group by tissue type compared to the whole proteome and the mitonuclear proteome (see “Methods”, Supplemental File 5). Pairwise statistical significance (Wilcoxon rank-sum test) of difference between each disease group and the whole proteome or mitonuclear proteome is indicated by *color of dot* in the *upper* and *lower right*-*hand corners*, respectively. **b** Example *box*-and-*whisker plot* of mitonuclear gene expression in cerebral cortex by disease gene group. *p < 0.05. **p < 0.005 by Wilcoxon rank-sum test

Mitochondria perform a wide range of functions that are of differential importance in different tissues, and tissue-specific roles of mitochondria undoubtedly contribute to the role of this organelle in disease. To explore tissue specificity of the expression of genes within each group, we used recently published RNA-seq data studying genome-wide gene expression in 32 different tissues (Uhlen et al. 2015), comparing the relative expression of genes in each group with both the whole proteome and the mitonuclear proteome (Fig. 3; Supplemental File 5, see “Methods”). We found that each disease gene set is associated with a unique tissue expression profile. Strikingly, the neurodegenerative disease gene set is specifically highly expressed in cerebral cortex, while the genes in the metabolic disease group show high expression in liver, intestines, and duodenum. In these diseases, the GWAS-identified genes appear to be specifically highly expressed in disease target tissues. By contrast, cancer, cardiovascular disease, and psychiatric disorders show similar expression throughout all 32 tissues. Thus, in some diseases expression of the disease-associated genes is enriched in known target tissues, while genes associated with other diseases, such as cancer, show no tissue overt specificity.

### Cancer mitonuclear GWAS provides novel insights into pathways of cancer risk

**Fig. 4:**
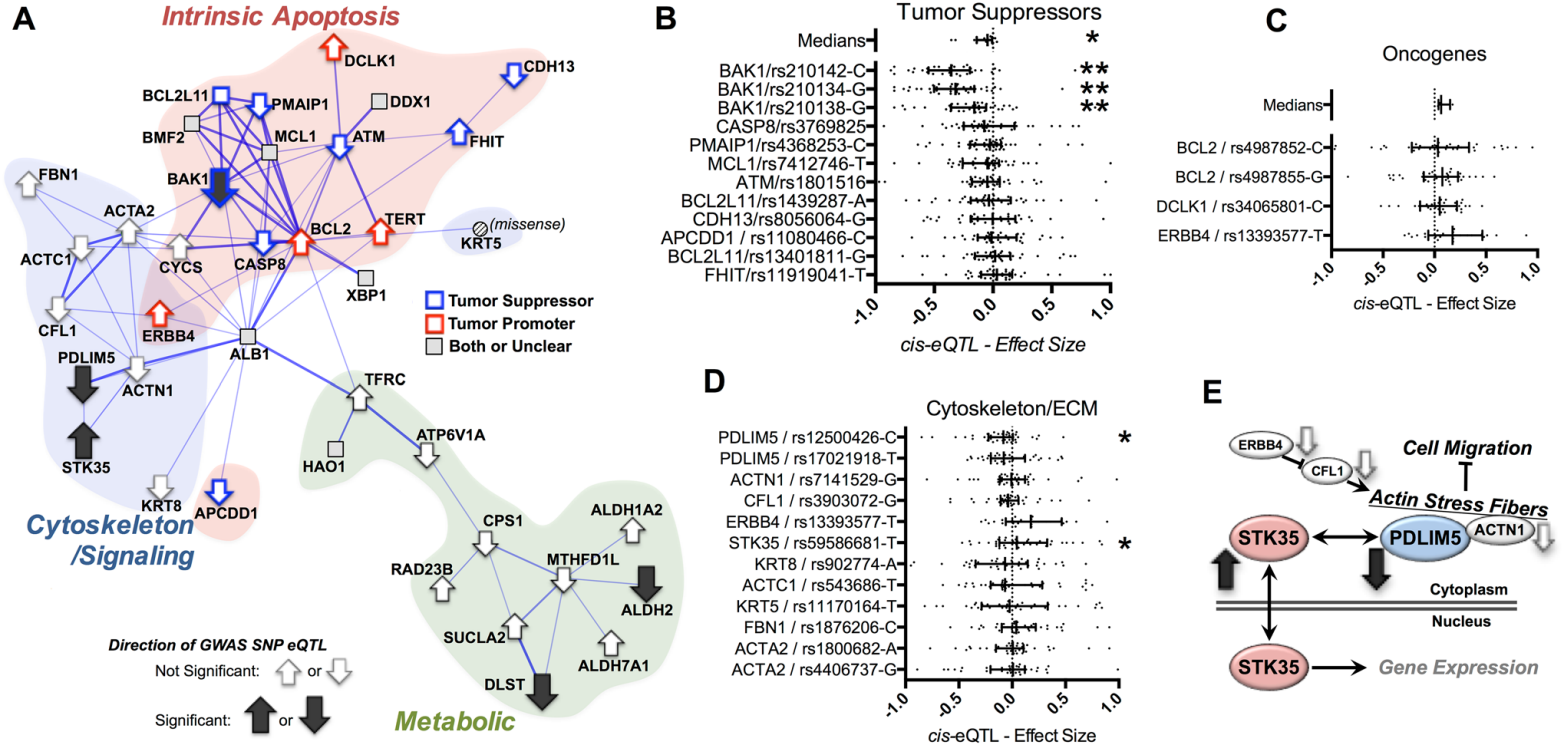
Mitonuclear cancer associations and eQTL data reveal directional impact of risk alleles on intrinsic apoptosis and intracellular signaling. **a** The cancer mitonuclear GWAS network includes three overlapping sub-networks: intrinsic apoptosis, cytoskeleton/signaling, and metabolic. Among the intrinsic apoptosis factors, tumor promoters and tumor suppressors are indicated in red and blue, respectively. **b**, **c** eQTL data for tumor suppressors and oncogenes, respectively. Direction of eQTL effect is overlaid on **a** using *arrows*. **d** eQTL data for cytoskeletal/signaling genes in the cancer network (direction and significance overlaid in **a**). **e** A putative model for the relationship between the cytoskeletal/signaling network and cancer risk. *p < 0.05, **p < 0.005 by one-sample *t* test (see Supplemental Data)

Given the striking enrichment of the intrinsic apoptotic pathway in the cancer network, we decided to examine this group in greater depth. The STRING network for cancer can be divided into three sub-network clusters: a cluster of apoptosis genes, a cytoskeleton/signaling cluster, and a metabolic cluster (Fig. 4a red, blue, and green regions, respectively). To investigate whether the GWAS-identified SNPs are associated with altered gene expression, which might functionally impact the integrity of these sub-networks, we examined the variants by gene-level cis expression quantitative trait loci (eQTLs) analysis and determined the directional effect of risk alleles using the Genotype-Tissue Expression (GTEx) database (http://www.gtexportal.org) (Carithers and Moore 2015) (see “Methods”). We divided the genes involved in the ‘apoptosis’ cluster into ‘tumor suppressors’ and ‘tumor promoters’ based on their known pro- or anti-apoptotic effects, respectively. Strikingly, eQTL analysis reveals that GWAS risk alleles in ‘tumor promoters’ are associated with increased expression while ‘tumor suppressors’ are associated with decreased expression or no change (Fig. 4a–c). While only three BAK1 SNPs and the pooled medians for pro-apoptotic genes reach statistical significance, the directionalities of the remaining gene–SNP pairs are consistent with reduced pro-apoptotic signaling (see also Supplemental File 6).

Among genes in the cytoskeleton cluster, two *cis*-eQTLs were significant: PDLIM5 (PDZ And LIM Domain protein 5) and STK35 (serine–threonine kinase Clik1, also known as PDLIM interacting kinase 1), with the cancer risk alleles associating with significantly decreased and increased expression, respectively (Fig. 4d). STK35/Clik1 regulates gene expression, while the PDZ-LIM family comprises stress fiber-associated proteins that control regulatory factors through sequestration in the cytoplasm; at least one PDLIM family member has been shown to directly target STK35 to stress fibers (Vallenius and Makela 2002). The other gene products in the cytoskeletal cluster, CFL1 (cofilin 1), ACTN1 (alpha actinin 1), and ERBB4 (encoding receptor tyrosine protein kinase erbB-4), all interact to regulate actin stress fibers (Hirata et al. 2015; Opitz et al. 2015). Moreover, the STK35 homologue STK35L1 is a known regulator of the tumor suppressor p16(INK4a) (Goyal et al. 2011). Thus, the directional effects of the risk alleles on expression of STK35 or PDLIM5 are consistent with increased cancer risk (Fig. 4e).

## Discussion

**Fig. 5:**
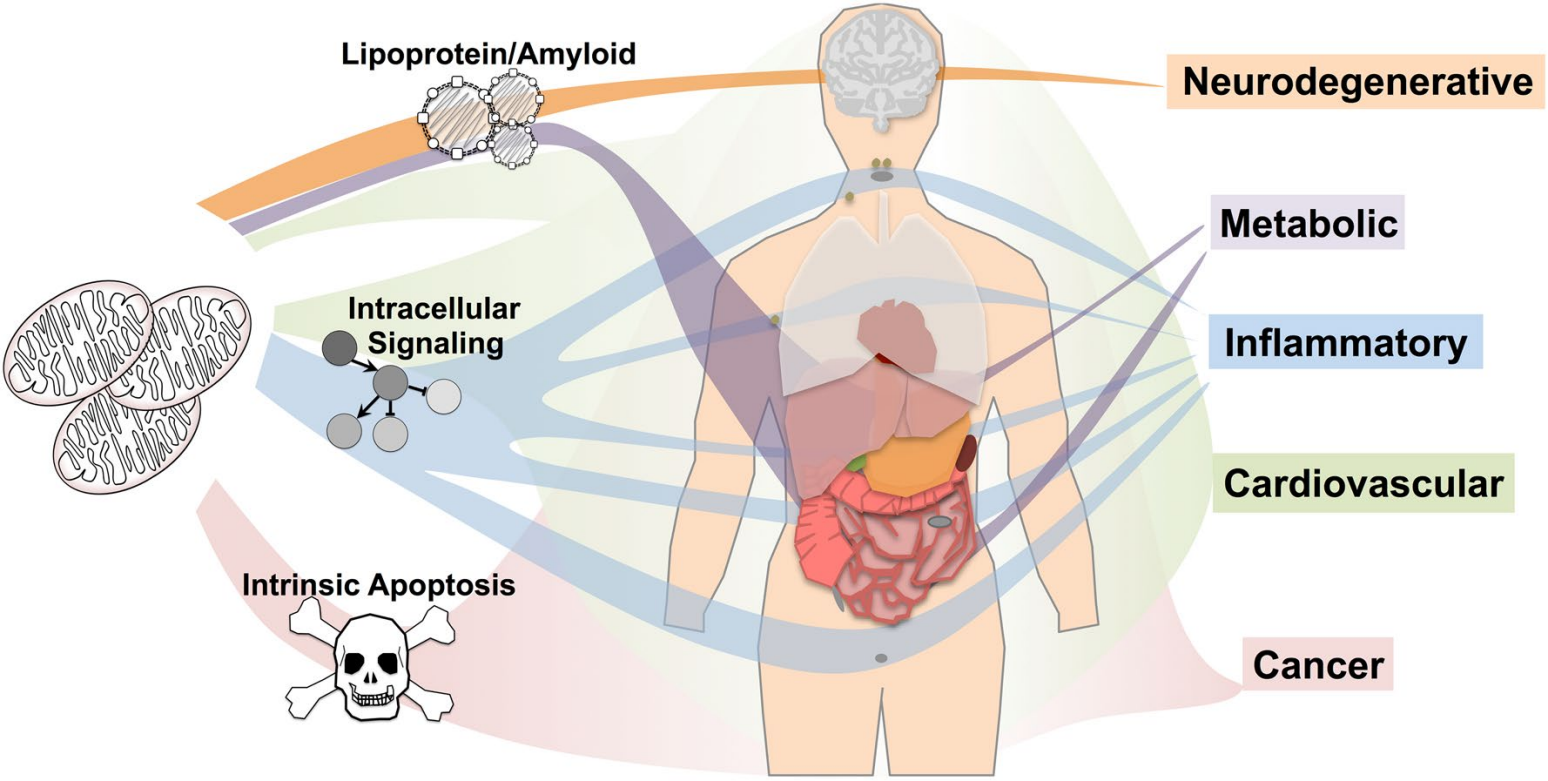
The role of common genetic variation in mitonuclear genes in human disease. Network and pathway analysis of mitonuclear GWAS and genome-wide expression data reveals unique pathways and tissue expression patterns underlying mitonuclear gene–disease associations. Mitonuclear cancer-associated genes are enriched for intrinsic apoptosis and broadly expressed. Cardiovascular disease mitonuclear genes are enriched for lipoprotein metabolism with no tissue showing significantly higher expression than the background. Inflammatory disease is enriched for signaling genes, with high expression in lymph, spleen, tonsil, appendix, and other tissues. Metabolic and neurodegenerative mitonuclear genes are enriched for lipoprotein/amyloid processes but show differential tissue expression: metabolic disease genes are highly expressed in liver and intestines while neurodegenerative disease genes are highly expressed in brain

Using unbiased assessment of GWAS, RNA sequencing, and eQTL datasets, we have identified unique disease-specific protein interaction networks, functional pathways, and tissue-specific expression patterns of mitonuclear genes (Fig. 5). In cancer, GWAS risk genes are primarily enriched for one process, intrinsic apoptosis, and are broadly and systemically expressed. Similarly, cardiovascular disease is enriched for one pathway, lipoprotein metabolism, with no apparent tissue-specific expression. Inflammatory disease is enriched for intracellular signaling genes, especially in response to stress, highly expressed in multiple tissues including lymph nodes, spleen, tonsil, and appendix. Metabolic disease and neurodegenerative disease genes are both enriched for lipoprotein/amyloid processes but show differential tissue expression: metabolic disease-associated genes are highly expressed in liver and intestines while neurodegenerative disease genes are highly and specifically expressed in the brain. As a whole, these data indicate that while mitochondrial dysfunction is ubiquitously linked to a wide range of pathologies in humans, the landscape of common genetic variation among mitonuclear genes is highly disease specific, as are the mechanistic relationships between mitochondria and disease both in regard to mitochondrial pathways and tissue-specific expression.

The striking differences in tissue expression between cancer, where there are no apparent tissue-specific expression profiles, and diseases such as neurodegenerative disease, which shows a highly tissue-specific expression pattern, are particularly interesting. With only tissue expression data these differences would be difficult to reconcile, but the pathway- and function-based analyses provide some clues. From these data, it appears that the mitochondrial pathways involved in cancer are highly evolutionarily conserved pathways likely to function in most or all human cell types, whereas the pathways of neurodegenerative disease, which include amyloid processing and lipoprotein metabolism, are more recently evolved and likely enact tissue-specific functions. Perhaps more interesting are the differences in expression profiles between neurodegenerative disease and metabolic disease, which show enrichment of overlapping pathways but unique tissue expression profiles among disease-associated genes. This indicates that genetic perturbations of genes involved in lipoprotein metabolism processes can lead to either neurodegenerative disease or metabolic disease, depending on where the perturbed factor is expressed (see Fig. 5). Together, these results provide some insight into the pathogenesis of complex genetic diseases and suggest that tissue targeting should be considered when developing therapeutic strategies.

The challenge of exploiting the ever-expanding GWAS catalog is to find functional insight into disease processes in seemingly disparate sets of genes. Here, we used network-based approaches to study the GWAS mitonuclear genes in bulk. Our results provide new insight into the relationship between mitochondria and human disease and open new avenues for research. The cancer intrinsic apoptosis eQTL findings demonstrate that the directional impact of risk variants on gene expression can predict target pathways for intervention. Pharmaceutical agents targeting the intrinsic apoptotic pathway are already in experimental and clinical use (Almstedt and Schmidt 2015; Mohana-Kumaran et al. 2014; Mukherjee et al. 2015; Opitz et al. 2015; Paraiso et al. 2012; Subramaniam et al. 2015; Yaswen et al. 2015), so it is readily conceivable that cancer patients could be screened for variants in this hub and treatment designed based on their genotype. In addition, our findings of tissue specificity in certain disease gene sets reinforces the importance of considering tissue specificity in drug screening and development, while also suggesting that interventions targeted to the tissue of interest may provide greater benefit with fewer off-target effects.

This study utilized both genetic association studies and experimental expression data but is limited by a lack of functional studies characterizing the impact of GWAS risk alleles on cellular and molecular endpoints beyond gene expression. Such studies will provide critical links between large-scale human genetic findings and disease pathogenesis, solidly tying these in silico findings to human biology. An additional caveat in our findings is that while our study was aimed at characterizing the role of genetic variation in mitonuclear genes in human disease, and most of the significant findings are indeed bona fide mitochondrial pathways, many specific genes and identified do not have obvious direct mitochondrial roles. The composite mitonuclear gene list was intended to be comprehensive and contains any genes whose products have been reported to localize to mitochondria. Thus, while the findings reported here are comprehensive, specific factors may be only loosely relevant to mitochondrial biology. While generation of a comprehensive and accurate mitochondrial proteome is an active area of research and our study is limited by available data, we cross compared the protein interaction networks in Fig. 2 with the cellular localization database COMPARTMENTS (see “Methods”) to provide additional context for our findings (Binder et al. 2014). The COMPARTMENTS database assesses the strength of evidence showing localization of proteins to cellular compartments, including mitochondria, by distilling multiple localization resources into a simple score, where higher scores (maximum 5) indicate stronger evidence for mitochondrial localization. This comparison revealed that highly connected factors in the network maps tend to show strong experimental evidence supporting mitochondrial localization, suggesting that even if a more stringent localization cutoff were available the key findings would be unchanged (Fig. S3).

Finally, it is important to emphasize that only the mitonuclear genome was examined, as no mtDNA-encoded genes appear among GWAS catalog disease trait associations. Properties of the mitochondrial genome such as copy number variance and heteroplasmy, the presence of multiple forms of mtDNA in the same cell or organism, necessitate distinct methodologies. Although encoding less than one percent of mitochondrial proteins, the proteins encoded are key to core mitochondrial function, and the impact of genetic variation in the mitochondrial genome warrants further study.

## Methods

### Mitochondrial proteome and mitonuclear GWAS compilation

Datasets to compile the genes encoding the mitochondrial proteome were downloaded during July, 2015, from MitoCarta (downloaded from http://www.broadinstitute.org/node/7098/index.html) (Pagliarini et al. 2008), the Integrated Mitochondrial Protein Index (IMPI) (downloaded from http://www.mrc-mbu.cam.ac.uk/impi) (Smith and Robinson 2015), and the Gene Ontology (GO) Mitochondrion catalogue (downloaded from MitoMiner at http://mitominer.mrc-mbu.cam.ac.uk) (Ashburner et al. 2000; Smith et al. 2012; Smith and Robinson 2015). The NHGRI GWAS catalog was downloaded in July 2015, and is available at https://www.ebi.ac.uk/gwas/ (Welter et al. 2014).

During submission of this manuscript an updated MitoCarta, MitoCarta 2.0, was published (Calvo et al. 2016). While the new MitoCarta contains approximately 20 % more proteins that the original, most of these factors were already present in our comprehensive mitochondrial proteome lists. MitoCarta 2.0 would add only 26 genes to our total gene set, only 15 of which are associated with disease traits by GWAS, additions that would not have substantially impacted our results. These are provided in Supplementary File 7.

### Statistical assessments

Statistical significance of GWAS association enrichment among mitonuclear genes was calculated by Chi-square test using GraphPad Prism. Overlap of genes within each disease group was statistically assessed by hypergeometric distribution using the R script dyper, part of the R Stats package (https://stat.ethz.ch/R-manual/R-patched/library/stats/html/Hypergeometric.html). Enrichments of protein–protein interactions and GO terms among disease-associated gene sets were calculated using the web-based interface for the STRING network database (http://string-db.org/). All enrichments were calculated using the gene list in the mitonuclear gene GWAS disease set as the background gene list, the most stringent method, unless stated otherwise.

### STRING networks

Protein–protein interaction networks were generated and assessed using the web-based Search Tool for the Retrieval of Interacting Genes/Proteins (STRING) pathway analysis tool (http://string-db.org/) (Szklarczyk et al. 2015). Networks presented in figures are curated to omit targets with no interactions (isolated from the network) and to overlay the number of GWAS catalog appearances for each gene in each disease group.

### Assessment of tissue-specific gene expression

Tissue-specific expression was examined using the recently published genome-wide RNA sequencing data from 32 human tissues (Uhlen et al. 2015). Pairwise comparisons between each group of genes and either the whole-transcriptome background or the mitonuclear gene set transcript background were performed using the Wilcox rank-sum test. Box plots represent Log2 Fragments per Kilobase of exon Per Million fragments Mapped (FPKM) +0.25. Bold bars represent median values, while the box spans the first to third quintile. Whiskers show minimum and maximum values with outliers, defined as ≥3 interquartile ranges above the third or below the first quartile, excluded.

### Assessment of GWAS variant eQTL directionality and significance

eQTL data in Fig. 4 were extracted from the GTEx Portal database (http://www.gtexportal.org/home/). Data in scatter plots represent eQTL data from all available tissues with directionality relative to GWAS risk allele. Data were analyzed and plotted using GraphPad Prism, with bars indicating interquartile range and statistical significance assessed using the two-tailed, one-sample *t* test method.

### R scripts and gene expression files

Script, text file descriptions, and gene expression files have been uploaded to the repository GitHub under the following URL: https://github.com/qwzhang0601/Network_analysis_of_mitonuclear_GWAS_gene_expression_in_tissues.

### Assessment of mitochondrial localization using compartments

To cross-examine evidence for mitochondrial localization of the factors involved the protein–protein interaction networks in Fig. 2, we queried the cell compartment localization database COMPARTMENTS (Binder et al. 2014) using the available web-based tool (http://compartments.jensenlab.org/Search). Values for each factor in Fig. 2 are indicated in Figure S3. For our purposes, scores of 0 and 1 were combined to a score of 1 as each factor in these networks appeared in at least one mitochondrial proteome dataset to appear in our analyses.

## Electronic supplementary material

Below is the link to the electronic supplementary material.

Figure S1 Additional comparative assessment of mitonuclear disease gene groups. A) Percent of genes overlapping between each of the disease groups. B) Number and percent of genes overlapping between each disease group in heat-map format with values included. C) Hypergeometric distribution *p* values of overlap between disease groups (TIFF 2512 kb)

Figure S2 Additional STRING network data. A) STRING protein–protein interaction network for psychological disease. B) STRING protein–protein interaction network for infectious disease. C) No association between enrichment of protein–protein interaction (p-value of observed vs. expected enrichment) and gene number in each group. D) Number of network interactions for genes appearing once or more than once in GWAS for psychological disease and infectious disease (TIFF 858 kb)

Figure S3 Mitochondrial localization score from COMPARTMENTS. Mitochondrial localization scores of factors in protein–protein interaction networks from COMPARTMENTS (see Methods) (TIFF 8704 kb)

Supplementary material 4 (XLSX 157 kb)

Supplementary material 5 (XLSX 726 kb)

Supplementary material 6 (XLSX 54 kb)

Supplementary material 7 (XLSX 1585 kb)

Supplementary material 8 (XLSX 39 kb)

Supplementary material 9 (XLSX 136 kb)

Supplementary material 10 (XLSX 36 kb)
