# Supplementary figures and images for: Network analysis of mitonuclear GWAS reveals functional networks and tissue expression profiles of disease-associated genes
Source: Hum Genet. 2016 Oct 4;136(1):55–65. doi: 10.1007/s00439-016-1736-9 (PMC5214989; doi:10.1007/s00439-016-1736-9)

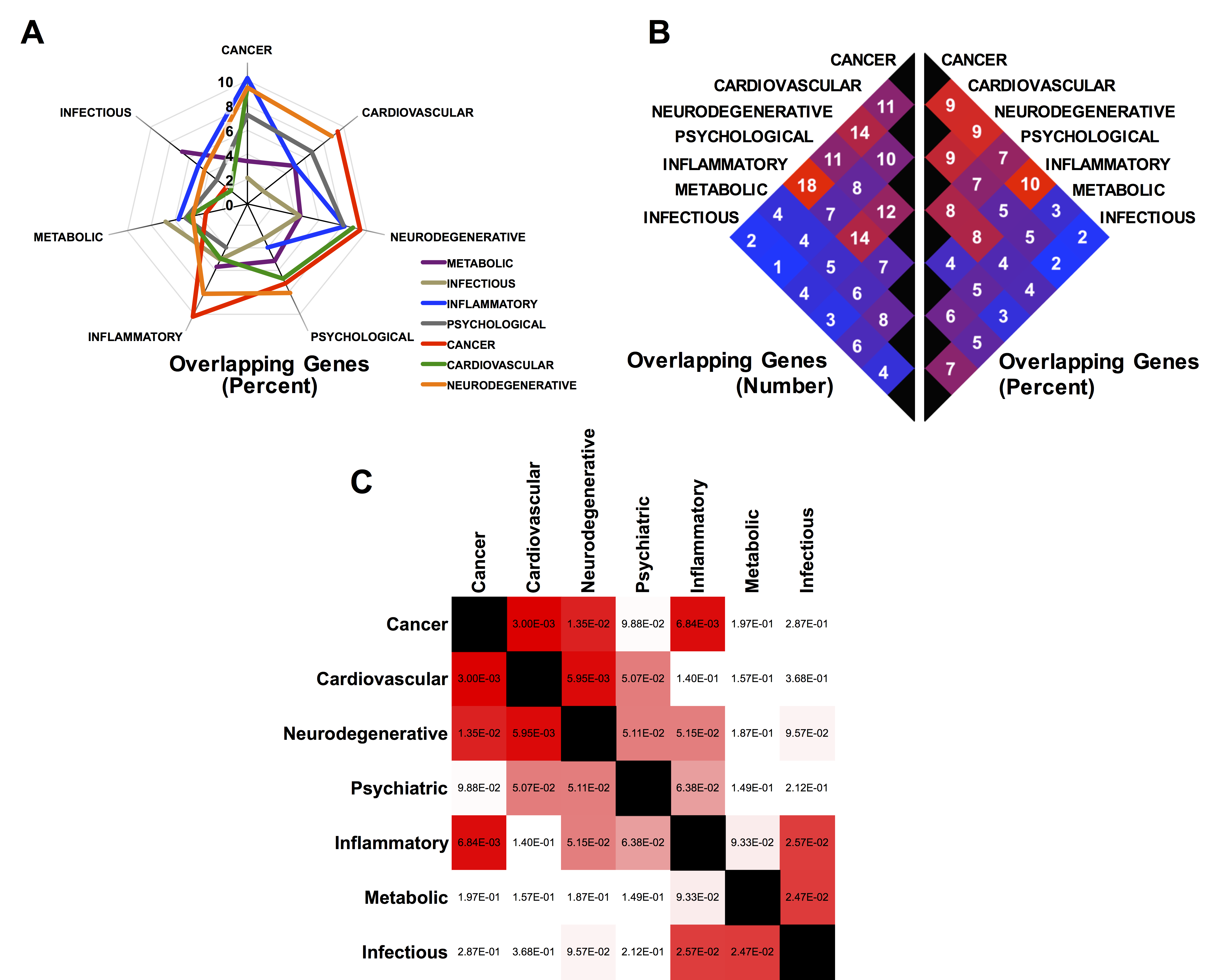

Supplement: Supplementary file 1 — Figure S1 Additional comparative assessment of mitonuclear disease gene groups. A) Percent of genes overlapping between each of the disease groups. B) Number and percent of genes overlapping between each disease group in heat-map format with values included. C) Hypergeometric distribution p values of overlap between disease groups (TIFF 2512 kb) [file 439_2016_1736_MOESM1_ESM.tiff]

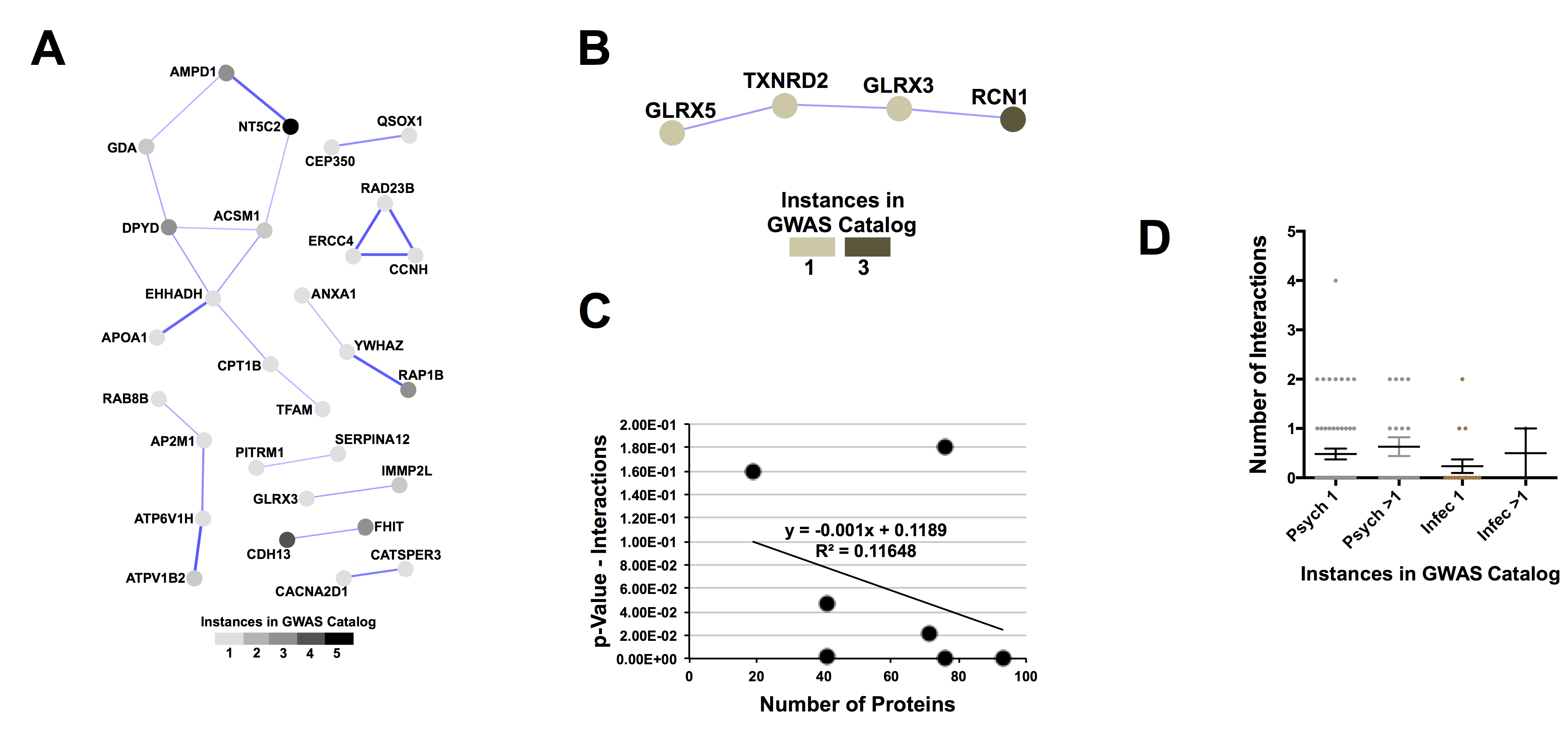

Supplement: Supplementary file 2 — Figure S2 Additional STRING network data. A) STRING protein–protein interaction network for psychological disease. B) STRING protein–protein interaction network for infectious disease. C) No association between enrichment of protein–protein interaction (p-value of observed vs. expected enrichment) and gene number in each group. D) Number of network interactions for genes appearing once or more than once in GWAS for psychological disease and infectious disease (TIFF 858 kb) [file 439_2016_1736_MOESM2_ESM.tiff]

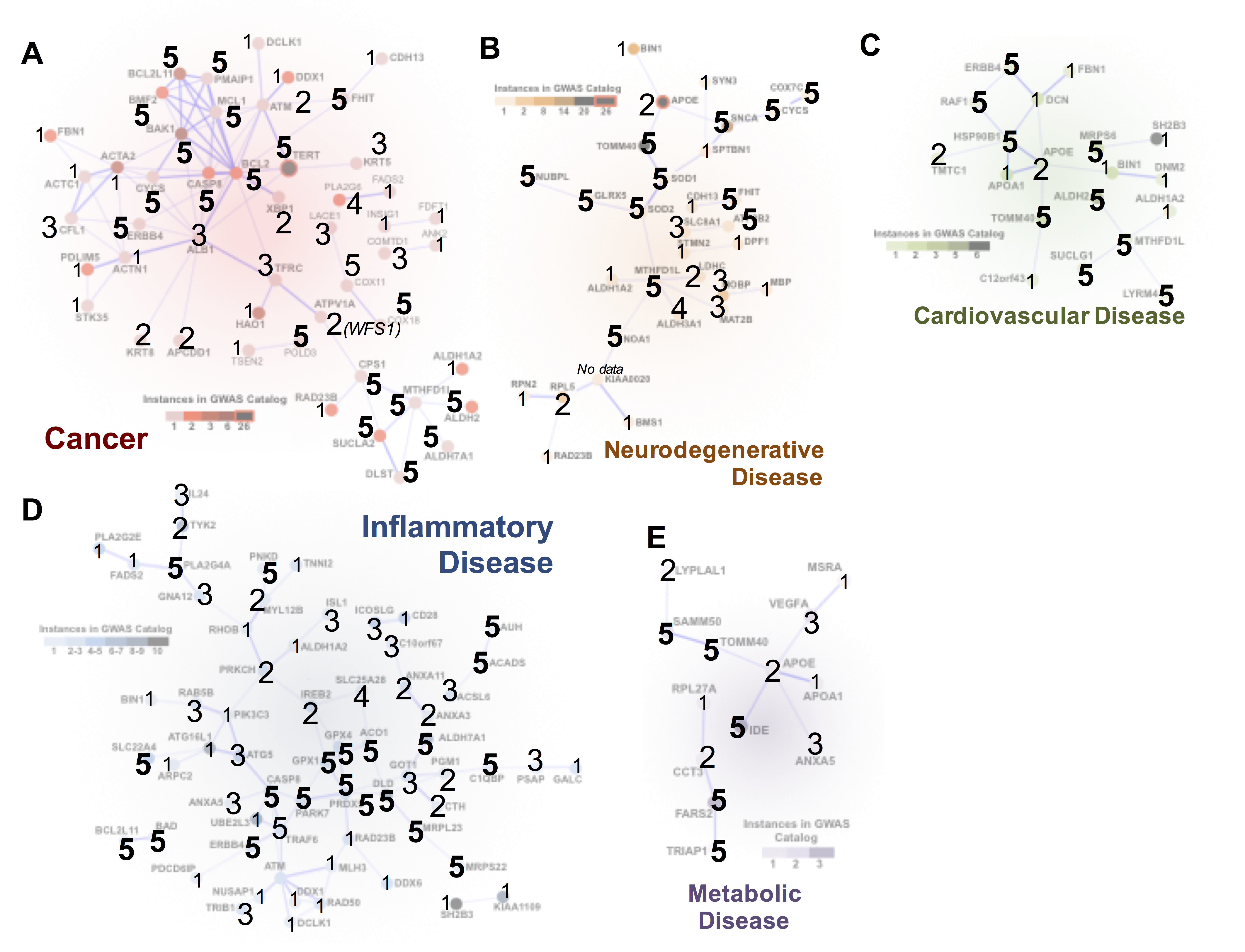

Supplement: Supplementary file 3 — Figure S3 Mitochondrial localization score from COMPARTMENTS. Mitochondrial localization scores of factors in protein–protein interaction networks from COMPARTMENTS (see Methods) (TIFF 8704 kb) [file 439_2016_1736_MOESM3_ESM.tiff]
